# Novel tumor-associated macrophage populations and subpopulations by single cell RNA sequencing

**DOI:** 10.3389/fimmu.2023.1264774

**Published:** 2024-01-29

**Authors:** Juanjuan Wang, Ningning Zhu, Xiaomin Su, Yunhuan Gao, Rongcun Yang

**Affiliations:** ^1^ Translational Medicine Institute, Affiliated Tianjin Union Medical Center of Nankai University, Nankai University, Tianjin, China; ^2^ Department of Immunology, Nankai University School of Medicine, Nankai University, Tianjin, China; ^3^ State Key Laboratory of Medicinal Chemical Biology, Nankai University, Tianjin, China

**Keywords:** tumor associated macrophages, cancer, single cell RNA sequencing, angiogenesis, metastasis

## Abstract

Tumor-associated macrophages (TAMs) are present in almost all solid tumor tissues. 16They play critical roles in immune regulation, tumor angiogenesis, tumor stem cell activation, tumor invasion and metastasis, and resistance to therapy. However, it is unclear how TAMs perform these functions. With the application of single-cell RNA sequencing (scRNA-seq), it has become possible to identify TAM subpopulations associated with distinct functions. In this review, we discuss four novel TAM subpopulations in distinct solid tumors based on core gene signatures by scRNA-seq, including *FCN1* ^+^, *SPP1* ^+^, *C1Q* ^+^ and *CCL18* ^+^ TAMs. Functional enrichment and gene expression in scRNA-seq data from different solid tumor tissues found that *FCN1* ^+^ TAMs may induce inflammation; *SPP1* ^+^ TAMs are potentially involved in metastasis, angiogenesis, and cancer cell stem cell activation, whereas *C1Q* ^+^ TAMs participate in immune regulation and suppression; And *CCL18* ^+^ cells are terminal immunosuppressive macrophages that not only have a stronger immunosuppressive function but also enhance tumor metastasis. *SPP1* ^+^ and *C1Q* ^+^ TAM subpopulations can be further divided into distinct populations with different functions. Meanwhile, we will also present emerging evidence highlighting the separating macrophage subpopulations associated with distinct functions. However, there exist the potential disconnects between cell types and subpopulations identified by scRNA-seq and their actual function.

## 1 Introduction

Macrophages, which exist in almost all tissues and organs, not only contribute to immune regulation, tissue regeneration and remodeling (1) but also play critical roles in the occurrence and development of diseases (2–4). TAMs are important immune cells in the tumor microenvironment (TME). They determine tumor growth, metastasis and prognosis (5–7). They are closely related to poor prognosis and resistance to therapy (8, 9). These cells include TAMs from embryo-derived TRMs and inflammatory monocytes (10, 11). TAMs from monocytes can increase with tumor growth due to self-proliferation, recruitment, and differentiation from circulating inflammatory monocytes (7, 12), which are mediated by elevated secretion of cytokines by cancer and stromal cells in tumors and associated metastases (13–15).

Macrophages are divided into two different subpopulations, M1 and M2, based on *in vitro* culture (16–18). TAMs with M1- and M2-like phenotypes represent two extremes of TAM polarization. These TAMs display distinct functions in tumor tissues. M1-like TAMs, which express surface molecules such as CD68 and produce cytokines such as IL-1β, could act as the main forces in innate host defense; Whereas M2-like TAMs, which express immunosuppressive molecules such as CD163 and produce cytokines such as IL-10 and CCL18, are critical in promoting epithelial-mesenchymal transition (EMT), angiogenesis, and immunosuppression of tumors (7, 19, 20). However, according to present literatures, it is defective or even misleading for the M1/M2 dichotomy in cancer biology. Accumulating evidence has shown that some TAM subpopulations can express genes of both M1 and M2 macrophages (21). Studies have also found that TAMs have significant plasticity and heterogeneity, and are composed of multiple different subpopulations in TME (19). However, it is unclear how to distinguish these TAM subpopulations with different functions.

With the application of scRNA-seq, it has become possible to distinguish TAM subpopulations with distinct functions. scRNA-seq can not only discover relationships between the genes, and track the trajectories of different cell lineages, but also more importantly reveal different subpopulations, especially some rare cell populations. To distinguish TAM subpopulations with different functions, analyses can be conducted according to different compositions, functional enrichment, and differential gene expression.

One of the most frequently employed enrichment analysis tools for scRNA-seq data is DAVID website (http://david.niaid.nih.gov), which aims to provide a functional interpretation of large lists of genes derived from genomic studies (22). It includes the gene functional classification tool, functional annotation tool, gene ID conversion tool, gene name viewer, and NIAID pathogen genome browser (22). According to scRNA-seq data from different solid tumors in the current literatures, TAMs in solid tumor tissues can be mainly divided into four different kinds of TAMs, including *FCN1^+^*, *SPP1^+^*, *C1Q^+^* and *CCL18^+^* subpopulations. Here, we will review these macrophage subpopulations, which are related to the occurrence and development of tumors. The identified TAM subpopulations in tumor tissues can be potential prognostic biomarkers(s) and/or candidate therapeutic targets.

## 2 Origin of tumor-associated macrophages

With the application of scRNA-seq and modern lineage tracing techniques, a large body of evidence has shown that TAMs, which are derived from embryo-derived TRMs and inflammatory monocytes, can be found in tumors such as colorectal cancer, liver cancer, pancreatic cancer, lung cancer, and glioblastoma (23–25).

### 2.1 TAMs derived from embryo-derived TRMs

Recent data utilizing specific fate mapping technologies have provided evidence for the embryonic origin of tissue macrophages (26–28). These macrophages possess self-renewal and proliferation capacity. In most normal tissues, TRMs are mainly embryonic macrophages (29), which are necessary for the development of tissues and organs. Notably, solid tumors have requirements similar to those of developing organs and tissues in forming complex structures (30). Thus, macrophages in tumor tissues can also be derived from embryo-derived TRMs (31, 32). These TAMs from embryo-derived TRMs can contribute to the occurrence and development of cancers (33, 34).

### 2.2 TAMs derived from monocytes

In solid tumors, monocyte-derived macrophages (MDMs) are recruited by cytokines and chemokines and then polarized into TAMs, which are universally heterogeneous (13–15). MDMs can also self-renew and proliferate (35). For example, the proliferation of TAMs can be induced in the presence of granulocyte macrophage colony stimulating factor (GM-CSF) in liver cancer tissues (36). These monocyte-derived TAMs can switch from one phenotype to another. They display remarkable plasticity within the TME (37), which can result in distinct subpopulations. Tumor-associated factors, such as tumor hypoxia in the TME, contribute to the heterogeneity of monocyte-derived TAMs. Thus, monocyte-derived TAMs may consist of multiple subpopulations generated through distinct developmental pathways.

In addition, there is also a minor splenic contribution to monocyte-derived TAMs (38, 39). Although the bone marrow is the primary hematopoietic tissue and monocyte reservoir, the spleen is also an identified reservoir of monocytes, which can play a significant role in the inflammatory response (40). Thus, the spleen is also an important extra-medullary site that can continuously supply growing tumors with monocytes (39).

## 3 Novel TAM subpopulations in different solid tumors

**Figure 1:**
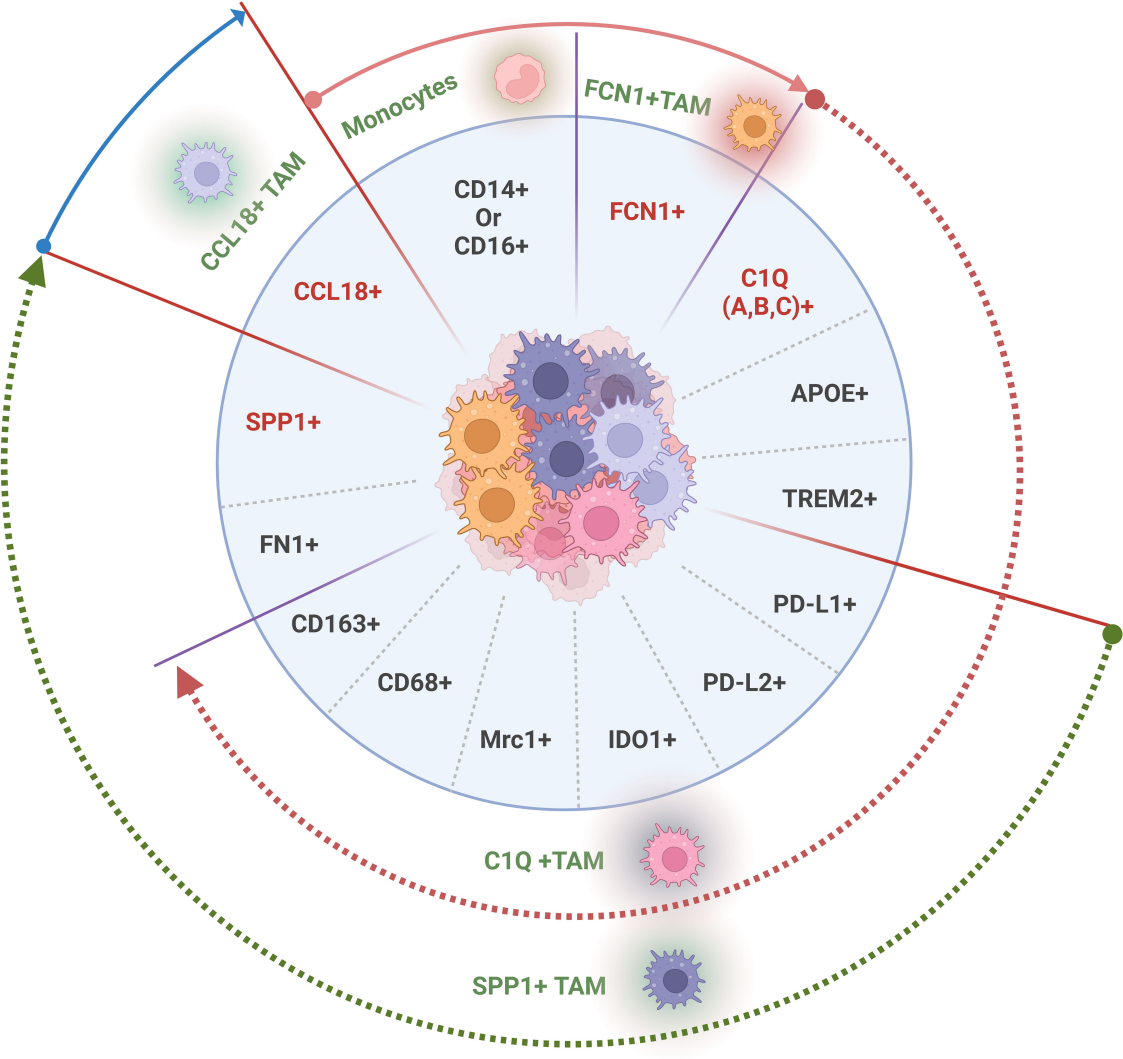
Tumor associated macrophage (TAM) subpopulations with distinct gene signatures in solid tumor tissues. The genes used to recognize TAM population and subpopulations, generally including T cell immune checkpoint ligands on the membrane surface such as PD-L1, PDL2, CD80, and CD86, surface immune suppressive molecules such as CD163, CD68 and MRC1 (CD206) and specific core gene signatures such as *FCN1* ^+^, *C1Q A/B/C* ^+^, *SPP1* ^+^ or *CCL18* ^+^ in different TAM subpopulations. Gene with red color, a core gene in each distinct TAM subpopulation.

There are many different gene signatures in TAM populations and their subpopulations. However, four classes of genes are generally used to recognize TAMs and their subpopulations, including macrophage-specific markers such as *CD14* and *MHCII*, T cell immune checkpoint ligands on TAMs, such as *PD-L1*, *PD-L2*, *CD80*, and *CD86* (41), surface immune suppressive molecules such as *CD163*, *CD68* and *MRC1 (CD206)* (42), and specific core gene signatures such as *FCN1*, *C1Q*, *SPP1* or *CCL18* in different TAM subpopulations. TAMs in different solid tumors are mainly divided into four subpopulations based on core gene signatures by scRNA-seq, including two main subpopulations *C1Q* ^+^ and *SPP1* ^+^TAMs, and two minor subpopulations *FCN1* ^+^ and *CCL18* ^+^ TAMs (**Figure 1**). Notably, there are differences in the gene expression in each TAM subpopulation in different tumors, the same tumors in different patients, and even different stages of the same tumor although there exists a core gene signature. ScRNA-seq studies have also demonstrated that these TAMs have high phenotypic plasticity and heterogeneity in cancers (43–45).

### 3.1 *SPP1* ^+^ TAM subpopulation

*SPP1* ^+^TAM subpopulation is identified by specific expression of a core gene *SPP1*. *SPP1* ^+^ TAM subpopulation also often expresses the following genes such as *FN1*, *IL1RN*, and other TAM genes *IDO*, *Mrc1(CD206)*, *PD-L1*, *PD-L2*, *CD68* and *CD163*. Notably, TAM associated *CD68* (46) and/or *CD206* (47) genes can be found only in the *SPP1* ^+^ TAM subpopulation of some solid tumors but not in all solid tumors (48). In addition, *SPP1^+^CD206* ^+^ TAM subpopulation also produces epithelial growth factor (EGF) (49).

*SPP1* ^+^TAM subpopulation was initially found in colorectal cancers (CRC) (43), and later in lung and breast cancers (50, 51). In human CRCs, *SPP1* ^+^ TAM subpopulations not only showed specific expression of *SPP1* but also *MARCO* and *VEGFA* (43, 52, 53). *SPP1* with glycolysis genes (*GAPDH*, *ENO1*, *LDHA*, *ALDOA*, and *TPI1*) was also expressed in TAMs in non-small cell lung cancer (NSCLC) (54). In lung squamous cell carcinoma, there also existed subpopulation of *SPP1* ^+^TAMs. *SPP1* ^+^ macrophages were significantly increased in the tumor microenvironment, which was related to the poor prognosis of patients with lung squamous cell carcinoma. *SPP1* ^+^ macrophages (53) were also found in hepatocellular carcinoma. Macrophage subsets that express *SPP1*, *TREM2* and *FN1* anti-inflammatory TAMs were found in breast cancer (8, 55, 56). The patients with *SPP1* ^low^ TAMs had the best prognosis for cervical cancer, whereas the worst prognosis appeared in patients with *SPP1* ^high^ TAMs (57). Multi-omics analysis also revealed the distinct clinical significance of *SPP1* ^+^ TAMs in cervical cancer (57). *SPP1* ^+^, TAM subpopulations could also be detected in rental cancer. and in pancreatic cancer (58).

### 3.2 *C1Q* ^+^ TAM subpopulation

The core gene signature of *C1Q* ^+^TAM subpopulation is the expression of *C1QA/B/C*. *C1Q* ^+^ TAMs are also characterized by the expression *PD-L1*, *PD-L2*, *HAVCR2*, *LGALS9*, and *CEACAM1* (52). PD-L1 and PD-L2, which are checkpoint molecules, can prevent CD8^+^ and CD4^+^ Th1 immunity (59). *C1Q* ^+^ TAMs can also highly express *TREM2*, *MERTK*, *CD80* (43, 57, 60), *SLC40A1*, *GPNMB* (43, 52, 61) and other genes in TAMs, such as *IDO*, *Mrc1* (*CD206*), *CD68* and *CD163*. In addition, this population of macrophages can also produce pro-angiogenic cytokines, such as vascular endothelial growth factor (VEGF).

A subpopulation of TAMs expressing *C1QA/B/C* is present in multiple cancers, such as colorectal cancer (43), NSCLC (62), liver cancer (44), lung cancer (63), rental cancer (60, 64, 65), breast cancer (8, 50), pancreatic cancer (58) and cervical cancer (57). Single-cell and spatial analyses revealed immunosuppressive phenotypes of *C1Q* ^+^ *APOE* ^+^ TAMs in CRCs (66). The patients with *C1QC^high^* TAMs had the best prognosis for cervical cancer, whereas the worst prognosis could appear in patients with *C1QC^low^* TAMs (57).

### 3.3 *FCN1* ^+^ TAM subpopulation

Another TAM subset, characterized by high expression of the core gene *FCN1*, is also identified in cancer tissues. This subpopulation of macrophages is a precursors of *C1Q^+^* TAM (43, 44). They are derived from inflammatory monocytes. Indeed, tumor-enriched *FCN1+* monocyte-like cells showed a high similarity to blood CD14^+^ monocytes, representing a monocyte population migrating into tumors and harboring a tumor-specific transcriptional program (43, 45).

*FCN1^+^* TAM subpopulation was also identified in human multiple solid cancers such as NSCLC (62), liver (44, 53), breast cancer (8), lung (45), CRCs (43) and pancreatic cancer (58). In human NSCLC, *FCN1* ^+^ macrophages, together with typical *FYN* ^+^ and *STAT1* ^+^ macrophages, expressed genes related to increased inflammatory function (62).

### 3.4 *CCL18* ^+^ TAM subpopulation

This population is characterized by the expression of a core gene *CCL18*. CCL18 is associated with the immunosuppressive nature of the tumor microenvironment. *CCL18* ^+^ TAM subpopulation is also an important element in cancer immune evasion (67). Notably, both *CCL18* ^+^ and *SPP1* ^+^ macrophages highly express immunosuppressive M2-like genes (62), implying that they are the same type of TAMs. *CCL18* ^+^ TAMs should be terminal *SPP1^+^* macrophages.

*CCL18* ^+^ TAM subpopulations can be found in many tumors such as NSCLC (62), breast cancer (68), CRCs (69, 70) hepatocellular carcinoma (53, 71), thyroid cancer (72) and intrahepatic cholangiocarcinoma (53).

Thus, four TAM subpopulations with different core genes such as *C1Q* ^+^, *SPP1* ^+^, *FCN* ^+^ and *CCL8^+^* TAM have been identified in distinct solid tumors by scRNA-seq data. However, there mainly are two TAM subpopulations in solid tumors, including *C1Q* ^+^ and *SPP1* ^+^ TAMs (43, 57, 73).

Notably, there also exist other classification methods based on single cell RNA sequences. For example, one study separated TAM into seven TAM subsets in tumor tissues, including inhibin beta A chain (INHBA)^+^ TAMs, complement C1q subcomponent subunit C (C1QC)^+^ TAMs, ubiquitin like protein ISG15 (ISG15)^+^ TAMs, NACHT, LRR and PYD domains containing protein 3 (NLRP3)^+^ TAMs, LYVE1^+^ TAMs, and sphingosine- 1-phosphate phosphatase 1 (SPP1)^+^ TAMs (74). TAMs were also separated into five TAM subsets in another study, including transcription factor HES-1 (HES1)^+^, complement component 1q (C1Q)^hi^, triggering receptor expressed on myeloid cells 2 (TREM2)^+^, IL4I1^+^ and proliferating TAMs (75). Other also showed seven TAM subsets in tumor tissues, including interferon-primed TAMs (IFN-TAMs), immune regulatory TAMs (reg-TAMs), inflammatory cytokine-enriched TAMs (inflam-TAMs), lipid-associated TAMs (LA-TAMs), pro-angiogenic TAMs (angio-TAMs), resident-tissue macrophage-like TAMs (RTM-TAMs) and proliferating TAMs (prolif-TAMs) (76).

## 4 Potential functions of novel TAM subpopulations

**Figure 2:**
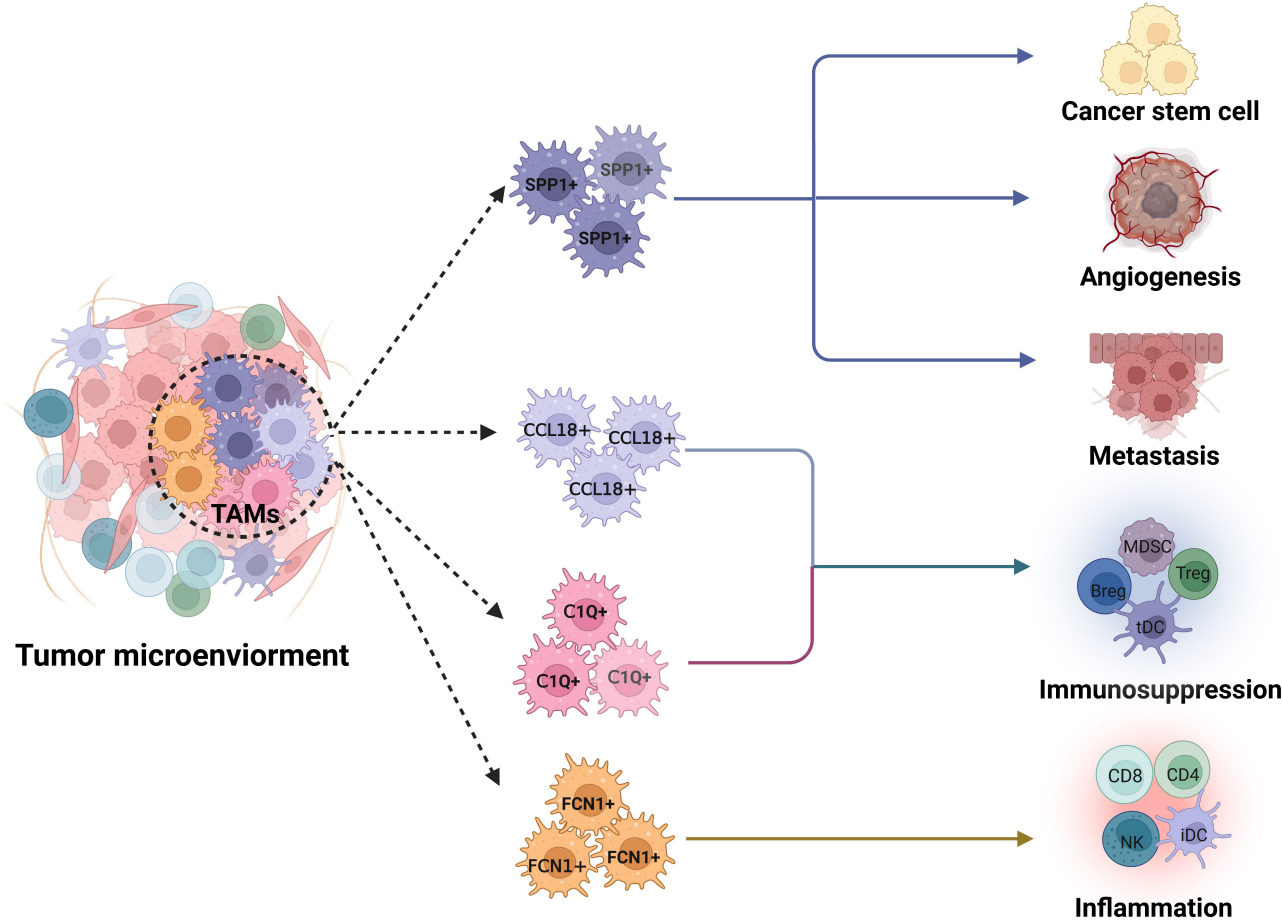
Potential functions of *FCN1* ^+^, *C1Q* ^+^, *SPP1* ^+^ and *CCL18* ^+^ TAM subpopulations in tumor microenvironments. *SPP1* ^+^ tumor associated macrophage (TAM) subpopulation exerts multiple roles in tumor development such as tumor metastasis, angiogenesis and tumor stem cell activation; Whereas a main function of *C1Q^+^* TAM population is immunosuppression. CCL18 TAM subpopulation has stronger immunosuppressive function. *FCN1* ^+^ TAM subpopulation is inflammatory macrophages, which can cause inflammation. Dotted line arrow, predicted TAM subpopulations based on core gene signatures.

TAMs are generally associated with a poor prognosis and treatment resistance (8, 9, 77, 78). These TAMs play different roles in the occurrence and development of tumors, such as immunosuppression, angiogenesis, metastasis, tumor stem cell activation, inflammation, antigen-presenting and phagocytes (5–7). TAM subpopulations with distinct functions can be identified through functional enrichment and differential gene expression in scRNA-seq data (**Figure 2**).

### 4.1 *SPP1* ^+^ TAM subpopulation

The number of *SPP1* ^+^ macrophages significantly increases in the tumor microenvironment, which is correlated with poor prognosis (46, 79–85). *SPP1* ^+^ TAM subpopulation has multiple roles in tumor development such as tumor metastasis (62, 86–91), angiogenesis (92–94), tumor stem cell activation and immunosuppression (95).

Tumor metastasis includes multiple steps, such as epithelial-to-mesenchymal transition, trans-endothelial migration, extracellular matrix remodeling, and formation of pre-metastatic niches. In the patients with tumors, the enrichment *SPP1* ^+^ TAMs was associated with worse overall survival (86, 96). *SPP1* ^+^ macrophages from tumor tissues showed high expression of MMP9, MMP12, MMP14, and MMP19, which could contribute to the degradation of the basement membrane for the invasion of tumor cells (82). There had also an interaction between *SPP1* and CD44 in *SPP1* ^+^ macrophages to promote metastasis. The interaction between *SPP1* ^+^ macrophages and tumor epithelial cells activated downstream genes to mediate the activation of NF-κB, PI3K/Akt, VEGF, uPA, and MMPs, which could promote endothelial cell proliferation. The interaction of *SPP1* ^+^ macrophages with *FAP* ^+^ fibroblasts also appeared in colorectal cancer to promote metastasis (97). In *SPP1* ^+^ macrophages, the main metabolic pathway was glycolysis, which could promote tumor metastasis via angiogenesis and matrix remodeling (62). *SPP1* ^+^ macrophages were predominant in liver metastasis (86) and also potential biomarker for early lymph node metastasis (89). Colorectal cancer metastases in the liver could establish immunosuppressive spatial networking between tumor-associated *SPP1* ^+^ macrophages and fibroblasts, which supported colorectal cancer growth in the immunosuppressed metastatic niche in the liver (98). Studies also found that *SPP1* ^+^macrophages were metastasis accelerators of colorectal cancer (99). They were often found in mesenteric lymph node (MLN) with metastases. *SPP1* ^+^ macrophages also promoted prostate tumor progression by increasing the incidence of prostate intraepithelial neoplasia (100).

For tumor angiogenesis*, SPP1^+^* TAMs can be acted as “angiogenic switch.” They produce cytokines, such as VEGFA, platelet-derived growth factor (PDGF) and angiopoietin, to promote angiogenesis (93, 101). *SPP1^+^* TAMs with high angiogenesis scores showed the expression of genes related to angiogenesis. There was also a strong enrichment of tumor angiogenesis and tumor vasculature pathways in *SPP1* ^+^ TAMs (43). Under the influence of hypoxia, *SPP1^+^* TAMs could develop a pro-angiogenic phenotype by directly upregulating angiogenic molecules, such as VEGF-A (102) and angiogenic modulators, such as matrix metalloproteinase (MMP)7 (103). These cells could also interact with endothelial cells to promote angiogenic functions (94).

Cancer stem cells (CSCs) are subsets of tumor cells that play a key role in tumorigenesis. *SPP1^+^* TAMs could regulate CSC activation (104–106). For example, *SPP1^+^* TAMs promoted tumor growth and proliferation by secreting collagen (107) and insulin-like growth factor-1 (IGF-1) (108).

*SPP1* ^+^ TAMs are also implicated in T cell suppression (97, 109), and play a role in immune evasion in the cancers such as colon cancer (52, 62). Upregulation of PD-L1 by SPP1 could mediate macrophage polarization and facilitate immune escape in lung adenocarcinoma (110). A dramatic increase in *SPP1* ^+^ TAMs was positively correlated with *FAP* ^+^ fibroblasts in CRC tissues, which could impair the immunotherapeutic effects (97). The patients with high SPP1 expression achieved less therapeutic benefit from an anti-PD-L1 therapy cohort (97). A tumor immune barrier (TIB) formed by the interaction of *SPP1* ^+^ macrophages and cancer-associated fibroblasts (CAFs) was related to immunotherapy efficacy. Disruption of the TIB structure by blocking SPP1 should be considered a relevant therapeutic approach to enhance the therapeutic effect of immune checkpoint blockage (ICB) in HCC (111).

SPP1, an osteopontin, from *SPP1* ^+^ macrophages is a multifunctional secreted phosphorylated glycoprotein (112). It is also present in other cells such as osteoblasts, fibroblasts, and in tumor cells (112). The correlations between levels of circulating SPP1 and/or increases in SPP1 expression on tumor cells had poor prognosis in many types of cancer (112). The interaction of SPP1 and FN1 in TAMs with certain integrins promoted tumorigenesis in CRCs (43). SPP1-CD44 signaling in the glioma perivascular niche also enhanced cancer stem cell phenotypes and promoted aggressive tumor growth via the γ-secretase-regulated intracellular domain of CD44 (113). SPP1 was a key gene in the lymph node metastasis and a potential predictor of poor prognosis in head and neck carcinoma (114). In addition, SPP1 is involved in resistance to chemoradiotherapy through the induction of EMT, autophagy, epigenetic alterations, aberrant glucose metabolism and reduction of drug uptake (79, 111, 115).

### 4.2 *C1Q* ^+^ TAM subpopulation

The main functions of *C1Q^+^* TAM subpopulation are immune regulation and immunosuppression (116–119). Indeed, TAMs with high *C1Q A/B/C*, *HLA-DR*, *APOE*, and *TREM2* expression have a classically immunosuppressive phenotype (65, 116). These TAMs exert their immunosuppressive effects through surface molecules, cytokines, and metabolites. Surface molecules such as PD-L1, PDL2, CD80, and CD86, and cytokines such as IL-10 from TAMs could induce the differentiation of CD4^+^ T cells to Tregs (iTregs) (120, 121). A metabolite of tryptophan mediated by indoleamine 2, 3-dioxygenase (IDO) in *C1Q* ^+^ TAMs also played an immunosuppressive function directly or via the aryl hydrocarbon receptor in Treg, NK, and DC cells (122). Arginine depletion in TAMs could cause “arginine starvation” in T cells to inhibit these cells (123). Increased levels of IDO also limited the proliferation of cytotoxic CD8 cells (124). *C1Q* ^+^ macrophages could effectively suppress T cells (65, 125, 126). In RCC, the presence of C1Q+ macrophages correlated with exhausted T cells, forming a dysfunctional immune circuit (65, 127).

Notably, *C1Q* ^+^ TAMs are also related to positive responses to ICB therapy in melanoma and lung-carcinoma patients (117–119). Indeed, C1Q^+^ cell density was correlated with inhibitory receptors PD-1 and LAG3 at the CD8^+^ T cell surface (128). These C1Q^+^ macrophages express additional immune checkpoint ligands, such as PD-L1, PDL-2 (127, 128) and others such as CD40L, CTLA4, LAG3, PD-1, and TIGIT (57). Thus, these *C1Q* ^+^ TAMs may be a beneficial population for clinical applications (86). Notably, patients with *C1Q^high^* and *SPP1^low^* TAMs had the best prognosis, whereas the worst prognosis appeared in the patients with *C1Q^low^* and *SPP1^high^* TAMs (57, 129). In lung cancer, increased CXCL-10 was described in *C1Q* ^+^ TAM, which was related with an enrichment of the transcription factors IRF1, IRF7, and STAT1 (62). Interestingly, IRF1 was correlated with STAT1, HLA-DR, PD-1, and LAG-3 in metastases of colorectal cancer (130). In addition, *C1Q* ^+^ TAMs were also involved in phagocytosis and antigen presentation (43).

*C1Q* ^+^ macrophages to exert their functions also depend on C1Q, a recognition molecule of classical complement pathway, which can bind to immune complexes or other activators in the tumor microenvironment. Indeed, C1Q could regulate human macrophage polarization via interactions with LAIR1 (131), and modulate the cytokine expression while they digested lipid proteins, causing an M2-like polarization (132). C1Q also directly regulated T cell phenotype through internalization, binding to mitochondria, and regulation of mitochondrial metabolism (133, 134). However, it is incompletely clear how it controls immune activation, tolerance and exhaustion. In addition, C1Q could also interact directly with endothelial cells (EC) to promote neoangiogenesis, via still unknown cell surface receptors (128, 135).

### 4.3 *FCN1* ^+^ TAM subpopulation

*FCN1* ^+^ TAM subpopulation comprises inflammatory macrophages. This subpopulation of macrophages is associated with increased inflammatory function (62). Enriched *FCN1* ^+^ TAMs were detected in tumor-adjacent tissues. These *FCN1* ^+^ TAMs were considered an intermediate stage from monocytes to tumor macrophages (136). They were associated with angiogenesis. Subsequently, these cells can produce *C1Q+* and *SPP1+* TAM (43). In addition, FCN1 (ficolin-1) from *FCN1* ^+^ TAMs can be as a novel macrophage infiltration-associated biomarker for the diagnosis of pediatric inflammatory bowel diseases (137).

### 4.4 *CCL18* ^+^ TAM subpopulation

The effects of *CCL18* ^+^ TAM subpopulation on tumor cells include tumor cell proliferation, migration induction, invasion, EMT, angiogenesis, and lymphangiogenesis (67). *CCL18* ^+^ macrophages can be found in solid tumor tissues (53, 71) and in an immunosuppressive state in tumor tissues (70). They have a stronger tumor-promoting role than *SPP1* ^+^ macrophages (53). Single-cell spatial transcriptomic analysis also identified highly metabolic *CCL18* ^+^ TAMs in colorectal liver metastasis sites.

In the tumor, CCL18 chemokine produced by *CCL18* ^+^ TAMs (67) was a marker of neoplastic diseases (67). Notably, elevated CCL18 levels in the serum and tumor are related to a worse prognosis in patients (53). Indeed, CCL18 is associated with immunosuppressive functions and cancer immune evasion. The CCL18 could recruit CD4^+^CD45RA^+^CD25^-^ naïve T cells into the tumor niche, and then differentiated into Treg cells, as shown in gastric cancer (138) and breast cancer (139). Notably, the importance of CCL18 in neoplastic processes mainly includes a signal transduction from PITPNM3 (one of CCL18 receptors) in CCL18-dependent migration, invasion, and epithelial- EMT cancer cells (67). Studies with human umbilical vein endothelial cells (HUVECs) showed that CCL18 could cause the VEGF-independent migration and tube formation of these cells (140). CCL18 also had an influence on the proliferation of cancer cells, but this effect was dependent on the type of tumor (67). In breast phyllodes tumor, CCL18 also participated in myofibroblast differentiation (141). Thus, CCL18 should be a potential therapeutic target for anti-cancer therapy. However, there is very few studies on the effects on tumor development through blocking the activity of CCL18.

## 5 Differentiation of novel TAM subpopulations

**Figure 3:**
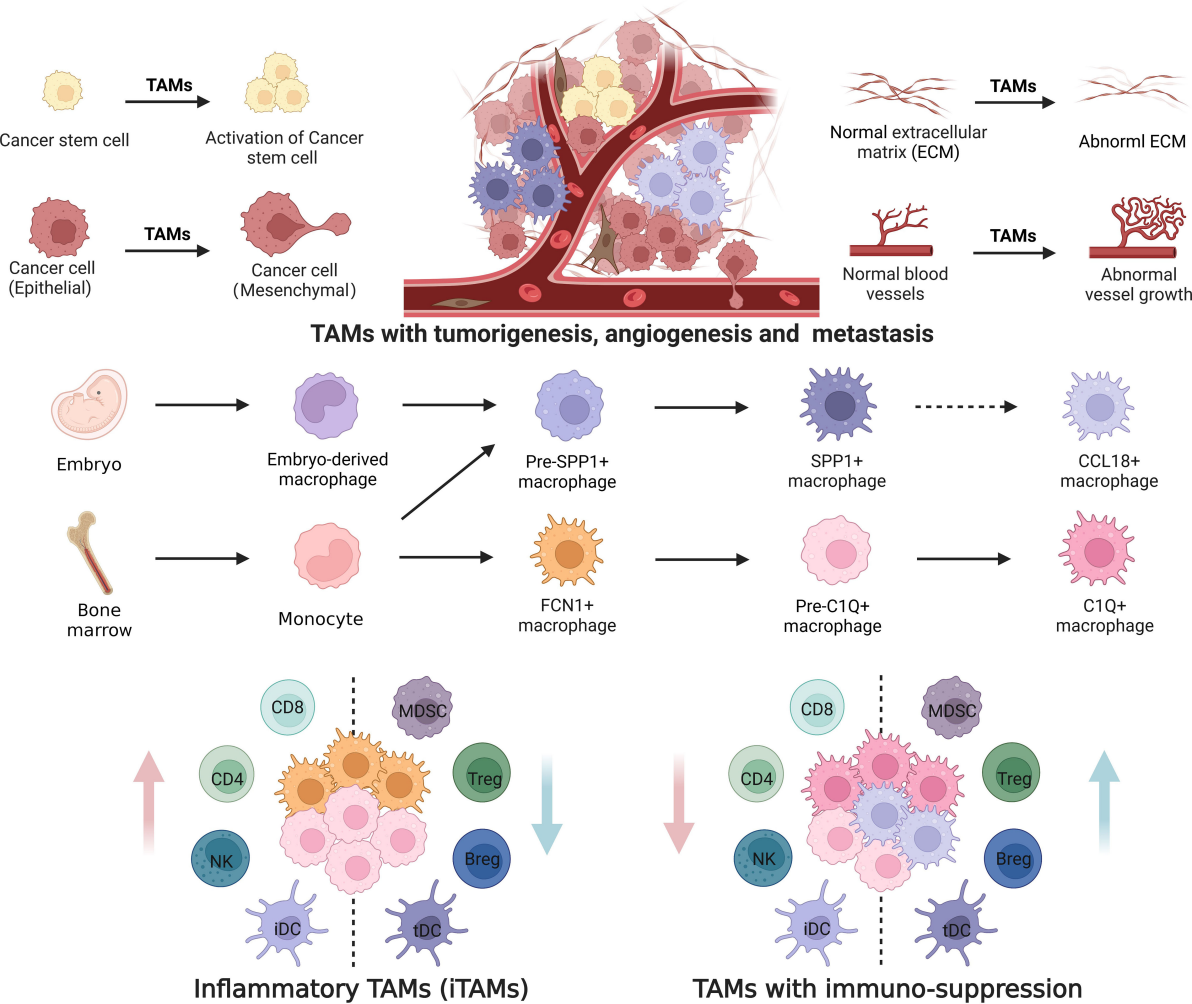
Differentiation of tumor associated macrophages with distinct functions. Tumor associated macrophages (TAMs) are both embryonic and monocyte origins. Monocytes derived TAMs highly express genes related to inflammatory and immunosuppression; While embryo-derived TAMs highly express genes related to tumorigenesis, metastasis and angiogenesis. *FCN1* ^+^ and *C1Q* ^+^ TAM subpopulations are derived from peripheral blood monocytes, whereas *SPP1* ^+^ and *CCL1*8^+^TAMs are derived from tissue-resident macrophages (pre-SPP1^+^ macrophages from embryo and monocyte derived macrophages). Differentiation of TAMs from peripheral blood monocytes to *C1Q* ^+^ macrophages includes monocytes, *FCN1+* macrophages, and pre-*C1Q* ^+^ and mature *C1Q* ^+^ macrophages. Differentiation of TAMs from tissue-resident macrophages into *CCL18* ^+^ macrophages occurs via pre-*SPP1* ^+^ and mature *SPP1* ^+^. Furthermore, mature *C1Q* ^+^ and *SPP1* ^+^ macrophages can be divided into different populations with distinct functions. iDC, inflammatory dendritic cells; tDC, tolerant dendritic cells; MDSCs, myeloid-derived suppressor cells; NK, natural killer cells. Dotted line arrow: predicted pathway based on gene expression.

TAMs have both embryonic and monocyte origins (142, 143). Genes related to immunosuppression and inflammation can be expressed by *FCN1* ^+^ and *C1Q* ^+^ TAMs (86, 87, 89, 97). Since monocyte-derived TAMs highly express genes related to inflammation and immunosuppression, *FCN1* ^+^ and *C1Q* ^+^ TAM subpopulations may be derived from monocytes. Whereas other two *SPP1* ^+^ and *CCL18^+^* TAM subpopulations express genes related to tissue remodeling, wound healing, metastasis and angiogenesis (67, 144). Notably, the genes related to tissue remodeling and wound healing also appear in embryo-derived TRMs (11, 24, 29, 142, 145). Thus, *SPP1* ^+^ and *CCL1*8^+^ TAMs might be derived from embryo-derived TRMs. Based on the scRNA-seq data, we suggest differentiation models of TAMs by tracking the development trajectories of distinct cell lineages (**Figure 3**). Monocyte-derived TAMs subsequently differentiate from peripheral blood CD14^+^ and/or CD16^+^-expressing monocytes to mature *C1Q* ^+^ macrophages through *FCN1+* inflammatory macrophages and pre-*C1Q* ^+^macrophages; Whereas the differentiation of TAMs from embryo-derived RTMs to *CCL18* ^+^ macrophages happens via pre-*SPP1* ^+^ and mature *SPP1* ^+^macrophages. Mature *C1Q* ^+^ and *SPP1* ^+^ macrophages can be further divided into the populations with distinct functions. However, others have also suggested that *FCN1*+ inflammatory macrophages can subsequently give rise to both *C1Q+* and *SPP1+* TAM populations (43).

### 5.1 From *FCN1* ^+^ to *C1Q* ^+^ *TAMs*

Monocyte subsets that highly expressed *FCN1*, *S100A8*,and *S100A9* could subsequently produce *C1Q* ^+^ TAM populations (43). *C1Q* ^+^ TAMs include pre-mature and mature *C1Q* ^+^ TAMs. Pre-*C1Q* ^+^ macrophages possess pro-inflammatory and phagocytic phenotypes based on the TAM differentiation routine. *C1Q* ^+^ and *FCN1* ^+^ macrophages with a pro-inflammatory phenotype could be detected in NSCLC (62). *C1Q* ^+^ TAMs with high phagocytic activity were also found in tumor tissues (125, 128). These TAMs highly also express genes involved in antigen presentation (43, 60). However, mature *C1Q* ^+^ macrophages are the predominant contributors to immunosuppression. These *C1Q* ^+^ macrophages express multiple immunosuppressive genes such as PD-L1, PD-L2, HAVCR2, LGALS9, and CEACAM1 (52). In addition, several different gene signature clusters in *C1Q* ^+^ TAM subpopulations have been reported, such as *C1Q* ^+^TAMs with genes *C1QA/B/C*, *SLCO2B1*, *NRP1*, *SLAMF8*, *FCGR1A*, *MERTK*, and *SIGLEC1* (43); *C1Q* ^+^TAMs with genes *C1QA/B*, *APOE*, *TREM2*, *GPNMB* and *SLC40A1* (44), *C1Q* ^+^TAMs with genes *TREM2*, *CD81*, *MARCO*, *APOE*, *CALR*, *CD63* and *SPP1* (63); and *C1Q* ^+^TAMs with genes *C1QB*, *APOE*, *FN1*, *CD276*, *TREM2*, *CHIT1*, *CCL18*, *MARCO*, *CD81* and *NRP2* (50). Since *C1Q* ^+^ TAMs mainly exert immunosuppressive functions, which are related to multiple immune cells, these TAMs with different gene signatures suggest the existence of multiple *C1Q* ^+^ TAM populations with distinct immunosuppressive functions (**Figure 3**).

### 5.2 From *SPP1* ^+^ to *CCL18* ^+^ TAMs

*SPP1* ^+^ macrophages not only promote tumorigenesis, but also metastasis and angiogenesis, which are possessed by embryo-derived TRMs (11, 24, 29, 142, 145), implying that this subpopulation is derived from embryo-derived TRMs. Several different gene signature clusters in *SPP1* ^+^ TAM subpopulations have been reported, such as *SPP1* ^+^TAMs with genes *SPP1*, *BCL6*, *ADM*, *MARCO*, *FN1*, *AQP9*, *TIMP1*, *VEGFA*, and *IL1RN* (57); *SPP1* ^+^ TAMs with genes *SEPP1*,*CD68*, *LYZ*, *MARCO*, *APOC1*, *SPP1* (82), *SPP1* ^+^ TAMs with genes *SPP1*, *MARCO*, and *VEGFA* (43, 52); and *SPP1* ^+^ TAMs with genes *SPP1*, *AQP9*, *TNS3*, *FN1*, *C15ORF48*, *PHLDA1*, and *NDRG1* (43). These TAMs with different clusters suggest that *SPP1* ^+^ TAM can be divided into different populations with distinct functions. In addition, *SPP1* ^+^macrophages possessed anti-inflammatory phenotypes (62), which played a role in immune suppression and tumor evasion in colon cancer (97, 109).

*CCL18* ^+^ macrophages belong to terminal TAMs. Increased immunosuppressive *CCL18* ^+^ TAMs with a terminally differentiated state and metabolically energetic phenotype could be found in tumors. *CCL18* ^+^ macrophages with high expression of CD163, MARCO, and CSF1R also exhibited stronger tumor-promoting effects than *SPP1* ^+^ macrophages (53). These macrophages with an anti-inflammatory phenotype could also induce a worse prognosis (53, 62). The effects of CCL18 on tumor cells were similar to *SPP1* TAM (67), implying that this *CCL8* ^+^ macrophage subpopulation was originated from *SPP1* ^+^ TAMs.

## 6 Relevance of TAM subpopulations by scRNA-seq with old M1 and M2 paradigm

Activated macrophages are usually divided into two categories, M1 and M2 (146). M1 is involved in pro-inflammatory responses, whereas M2 macrophages are mainly involved in anti-inflammatory responses. In tumor environments, M1 macrophages typically exert anti-tumor functions, including directly mediate cytotoxicity and antibody-dependent cell-mediated cytotoxicity to kill tumor cells; Whereas M2 macrophages can promote the occurrence and metastasis of tumor cells, inhibit T cell-mediated anti-tumor immune response, promote tumor angiogenesis, and lead to tumor progression (5). There are mainly two classes of TAM subpopulations identified by scRNA-seq in solid tumors, including *C1Q* ^+^ and *SPP1* ^+^ TAM subpopulations. *C1Q^+^* subpopulation in solid tumors possesses the characteristics of both M1 and M2 functions. This subpopulation is potentially involved in immunosuppression but also exhibits pro-inflammatory phenotypes. *C1Q^+^* TAM subpopulation (43, 44, 50, 63) also expresses common genes with both M1 and M2 macrophages (5, 20, 147). M2 TAMs are critical in promoting EMT, angiogenesis, and immunosuppression of tumors (7, 19, 20); Whereas *SPP1* ^+^ subpopulation can also potentially perform immunosuppression and mediate metastasis and angiogenesis although *SPP1^+^* TAM subpopulation also possesses other functions such as promotion of cell proliferation and tissue repair. This *SPP1^+^* TAM subpopulation also shares some genes with M2 macrophages (43, 50, 51). Thus, although *C1Q^+^* and *SPP1^+^* TAM subpopulations are different from old M1 and M2 paradigm, there also exist some common characteristics such as genes and functions between *C1Q^+^* and M1 TAM macrophages, and between *SPP1^+^* and M2 TAM subpopulations.

## 7 Tumor-associated macrophages as a target against tumor

TAMs, the most abundant immune cells in the TME, not only influence cancer progression and metastasis but also tumor recurrence (148). Thus, it is a critical strategy to target macrophages against tumor, which not only ameliorate the tumor-associated immunosuppression but also elicit anti-tumor immune responses. Different targeting strategies for TAMs have been developed, such as small molecular inhibitors and immune checkpoint inhibitors and antibodies.

The differentiation and function of macrophages can be manipulated by targeting PI3Kγ, JAK-STAT, C/EBPα, PPARγ and JNK1/2 signaling pathway using small-molecule inhibitors (149–151), and targeting immune-metabolism pathways such as arginine, adenosine, glutamine, tryptophan, kynurenine and lactate which have all been implicated in TAM reprogramming (152–154). Notably, STING (stimulator of interferon genes) could regulate the polarization of tumor-associated macrophages to inhibit liver metastasis of colorectal cancer (155). Targeting tumor-associated macrophages with STING agonist improved the antitumor efficacy of osimertinib in a mouse model of *EGFR-*mutant lung cancer (156). STING agonist overcomed STAT3-mediated immunosuppression and adaptive resistance to PARP inhibition in ovarian cancer (157). The activation of STING signaling also enhanced anti-tumor immunity (158), and improved cancer immunotherapy against tumor (159). In addition, epigenetic regulators such as histone deacetylases could also modify macrophage phenotypes (160, 161). However, these agents are not specific to macrophages, which have been associated with adverse toxicity.

Immune checkpoint inhibitors and antibodies which target TAMs can be used in therapy against tumors. TAMs and/or their subpopulations can overexpress different immune-checkpoint molecules such as PD-L1, which contribute to T cell exhaustion (162). Li et al. developed a hydrogel loaded with Pexidartinib and anti-PD-1-conjugated platelets to prevent of tumor recurrence after surgery (163). CD47 could interact with the inhibitory receptor SIRPα on the macrophages to transmit the “don’t eat me” signal (164). Several antibodies or small molecules targeting CD47-SIRPα axis have entered clinical trials (165, 166). A bispecific single-domain antibody was also used in the treatment of malignant tumors, which could efficiently and specifically bind and neutralize CCL2 as well as CCL5. They significantly induced the polarization of TAMs, and reduced immunosuppression in the TME (167). CD206 blockade also enhanced antitumor immune response in syngeneic models and the mouse pancreatic tumor model (168). Blockade of complement receptor C5aR1 also reprogramed tumor-associated macrophages (169).

In addition, other strategies such as depletion of TAMs and genetic engineering of macrophages with chimeric antigen receptor (CAR), which allows them to recognize tumor antigens and perform tumor cell-specific phagocytosis have also been used in therapy against tumors (170). One attractive target for TAM depletion is the CSF1-CSF1R axis (170). More than 30 phase I/II trials of small-molecule inhibitors or blocking antibodies to CSF1R have been initiated (149).

Notably, with the finding of new macrophage subpopulations by single cell RNA sequencing, more precise strategies which can target different TAM subpopulations will be developed. However, to the success of TAM subpopulation targeted therapies, it will be important to determine the exact functions of TAM subpopulations. Future studies should offer sufficient functional and phenotypic markers in the different TAM subsets. This will pave the way for the future development of targeted drugs to macrophage subsets. Notably, it may be difficult to study how embryo-derived TRMs and monocyte-derived TRMs influence tumors owing to limited consensus markers for these TRMs (149).

## 8 Conclusion and perspectives

TAMs and their subpopulations are related to tumor growth and progression. These TAMs and their subpopulations can act as potential new targets for cancer immunotherapy. Owing to the plasticity and heterogeneity of TAMs, it is difficult to define TAM subpopulations and their functions. However, scRNA-seq has served as a powerful tool for identifying these different TAM subpopulations. Here, we review subpopulations of TAMs in solid tumors in the phenotypic and transcriptomic levels, as well as their differentiation at the single-cell level. This review not only offers important insights into redefining subpopulations with different functions but also offers the possibility of precisely targeting TAM subpopulations. The application of scRNA-seq will undoubtedly promote our understanding of the biological characteristics of tumors.

However, there exists the potential disconnect between cell types and subtypes identified by scRNA-seq and their actual function. Thus, although scRNA-seq data have suggested TAM subpopulations with distinct functions, further clarification of these subpopulations with different functions is necessary for precise therapy and understanding of TAM characteristics. Next step will be to establish the actual function per TAM subpopulation. To determine the exact function of these TAMs, TAM population and subpopulation can be deleted through specific cytotoxicity or conditioned knockout.

## Author contributions

JW: Writing – original draft, Writing – review & editing. NZ: Writing – original draft, Writing – review & editing. XS: Supervision, Writing – review & editing. YG: Supervision, Writing – original draft. RY: Conceptualization, Funding acquisition, Supervision, Writing – review & editing.
